# Clinical Evaluation of Oral Midazolam Containing Cyclodextrin in Pediatric Magnetic Resonance: A Retrospective Cohort Study

**DOI:** 10.3390/jpm14050472

**Published:** 2024-04-29

**Authors:** Rossella Garra, Alessandra Piersanti, Miryam Del Vicario, Cecilia Maria Pizzo, Rossano Festa, Federica Tosi, Fabio Sbaraglia, Michelangelo Mario Spano, Filomena Della Sala, Marco Rossi

**Affiliations:** 1 Department of Anesthesia and Intensive Care, Fondazione Policlinico Universitario A. Gemelli IRCCS, Università Cattolica del Sacro Cuore, 00168 Rome, Italy; alessandra.piersanti@policlinicogemelli.it (A.P.); miryam.delvicario@policlinicogemelli.it (M.D.V.); rossano.festa@policlinicogemelli.it (R.F.); federica.tosi@policlinicogemelli.it (F.T.); fabio.sbaraglia@policlinicogemelli.it (F.S.); michelangelomario.spano@policlinicogemelli.it (M.M.S.); filomena.dellasala@policlinicogemelli.it (F.D.S.); marco.rossi@policlinicogemelli.it (M.R.); 2 Department of Anesthesia and Critical Care, Ospedale Pediatrico Bambino Gesù IRCCS, 00146 Rome, Italy; cmariapizzo@gmail.com

**Keywords:** oral midazolam, premedication, magnetic resonance imaging, general anesthesia, emergence agitation, emergence delirium, behavior change

## Abstract

Background: Reducing a child’s level of anxiety before magnetic resonance imaging (MRI) procedures allows for better behavioral outcomes. The aim of this retrospective study was to evaluate anxiolytic efficacy of Midazolam/γ-cyclodextrin oral formulation. Methods: We retrospectively reviewed 100 medical charts of children who, between 1 February and 31 July 2022, underwent MRI under general anesthesia with or without premedication with midazolam/γ-cyclodextrin. Primary outcome was comparison of behavior to facemask positioning, while secondary endpoints were degree of drugs acceptance, anxiolytic effect evaluation, child’s behavior on separation, and sevoflurane need. Results: Facemask positioning was accepted by 58% of the midazolam/γ-cyclodextrin group compared to 22% of children in the control group. The rate of acceptance was >90%. At the moment of separation from parent, none of the premedicated children needed to be restrained compared to 18% in the control group. A lower percentage of sevoflurane was needed for eye-closure at induction of anesthesia and for anesthesia maintenance. At emergence from anesthesia, 46% of children in the premedicated group compared to 66% of children in the control group showed transient agitation. Conclusions: Midazolam/γ-cyclodextrin showed a good profile of acceptance, satisfactory anxiolytic properties, and reduced need for anesthetics when administered to children before MRI under general anesthesia.

## 1. Introduction

Any diagnostic and therapeutic investigation performed on pediatric patients can lead to profound psychological discomfort [1,2,3], which is associated with poorer recovery, exacerbated pain perception [4], and overall difficulty in the child’s cooperation with self-care and adherence to treatment plans [5]. The unfamiliar environment, separation from parents, or simply fear of hospitalization is estimated to underlie stress and anxiety in over 50% of children during the periprocedural period [6].

Anxiety is an independent predictor for various behavioral changes that may encompass the following:

- Psychomotor agitation or delirium, typically occurring within 45 min from emergence of anesthesia and often resolving spontaneously in about 15–30 min [7];
- New maladaptive behaviors such as separation anxiety, crying, temper tantrums, and nightmares, from the first postoperative day (67%) up to two weeks (23%) or six months (20%) [8,9].

Kain et al. reported that children with marked emergence delirium (ED) had an odds ratio for developing new-onset postoperative maladaptive behavioral disorder of 1.43 compared to those without symptoms of ED [7].

Non-pharmacological interventions have been shown to relieve anxiety [10]. However, the difficulty of coping with the different etiological factors of anxiety often makes the use of drugs highly recommendable [11,12]. Midazolam is the most common sedative used in children due to its short half-life (around 120 min) and its myorelaxant and anterograde amnesia properties which facilitate avoidance of post-operative behavioral disturbances [13,14]. Over time, the intravenous formulation of midazolam has also been used for oral, nasal, or rectal administration, but those alternative routes of administration represent an off-label use [15]. Extemporaneous preparations obtained by mixing it with sweeteners (strawberry syrup, glucose 5%) help to mask the strong bitterness of midazolam while minimizing the tendency to spit out the dose, but, on the other hand, are not well controlled in terms of solubility and stability [16,17].

Thereby, an innovative, oral liquid formulation of midazolam/γ-cyclodextrin, ready to use in children from the age of 6 months, has received approval from the European regulatory agency in 2018 [18]. 

Midazolam/γ-cyclodextrin has been developed to align with the recommendations of the European Medicines Agency for pediatric drug palatability [19,20]: a sweetener sucralose and orange aroma were added for bitterness-masking effect and taste improvement while γ-cyclodextrin was used for its ability to solubilize lipophilic drugs [21,22].

In this retrospective study, we report our experience with the use of midazolam/γ-cyclodextrin protocol, focusing on the sedative and behavioral outcome in children who required magnetic resonance imaging (MRI) under inhalational anesthesia. 

## 2. Materials and Methods

This retrospective observational study adheres to the applicable Strengthening the Reporting of Observational Studies in Epidemiology (STROBE) guidelines. It was approved by the Internal Ethic Committee (ID Number: 5143, Protocol Number 0032517/22, on 14 October 2022) and it conforms to Good Clinical Practice and the Declaration of Helsinki. 

After the introduction of this formulation, in January 2022, our institution approved the supply of the drug and since then we began to implement a clinical management protocol for sedation/anxiolysis in the context of non-conventional services outside the operating room, focused on prevention of ED. 

A section specifically dedicated to this purpose was consequently added to the patient’s electronic medical record. Midazolam/γ-cyclodextrin became available in our hospital from May 2022.

Before its introduction, the majority of patients did not receive any premedication and only a small percentage underwent off-label administration of parenteral sedatives. 

**Figure 1:**
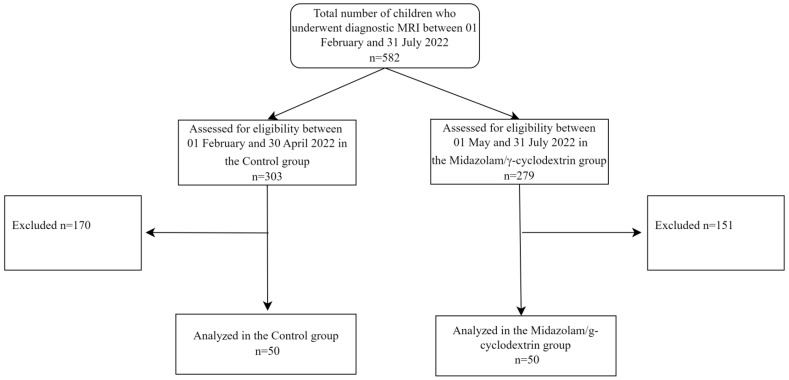
Flow diagram of the study participants. MRI: magnetic resonance imaging.

We performed a chart review from hospital medical records of all 582 consecutive children who, between 1 February and 31 July 2022, underwent diagnostic MRI at IRCCS Gemelli Foundation of Rome. Eligible patients were children aged ≥1 and ≤10 years, American Society of Anesthesiologists physical status (ASA) ≤3, who underwent the procedure under general anesthesia after receiving premedication with the recommended dose by the product leaflet of 0.25 mg/Kg midazolam/γ-cyclodextrin (Midazolam/γ-cyclodextrin group, May to July time period), or not (Control group, February to April time period) (Figure 1).

Patients’ charts were excluded according to the following criteria: age < 1 or >10 years, a history of hypersensitivity to midazolam or sevoflurane; chronic therapy with benzodiazepines and antiepileptics; acute respiratory tract infections; psychiatric and behavioral disorders; long term hospitalization over 3 months; American Society of Anesthesiologists physical status (ASA) > 3.

Of the 303 patients registered in the February–April period, 170 patients were not considered eligible, compared to 151 of the 279 children screened in the May–July period. For both the Midazolam/γ-cyclodextrin and Control groups, we enrolled the first 50 consecutive eligible patients in the study. 

In enrolled cases, all demographics, clinical and periprocedural data of interest were anonymously extracted by a keyword or manual search of electronic charts and recorded in an Excel file.

As for internal protocols, anesthesia management of children undergoing MRI is standardized. All children observe at least a 6 h period of pre-procedural fasting. Premedication is administered at the discretion of the anesthesiologist 30 min before induction of anesthesia. Parents of all children are present until transfer to the MRI suite. Anesthesia is induced with 70% nitrous oxide and 30% oxygen for 90 s, followed by incremental doses of sevoflurane in 0.5% steps, every minute, until eye closure, at a flow rate 2–3 times the minute volume through a Mapleson C circuit. The sevoflurane concentration is increased as needed and time of eye closure is recorded. Then, a face mask is positioned for maintenance of anesthesia through a Mapleson E circuit with fresh gas flows of 50% oxygen/air at a setting of sevoflurane percentage volume at the vaporizer, which is recorded. Standard monitoring includes EKG, SpO_2_, End-tidalCO_2,_ and inspired fraction of sevoflurane (Fi_sevo_), which is recorded at 5 min intervals until the end of MRI. In the Post-Anesthesia Care Unit (PACU), an active forced air warming is placed close to the patient and the parents are requested to stand by their child’ bedside. SpO_2_ is monitored and recorded at 5 min intervals until the child regains their state of wakefulness and consciousness and is discharged to the ward.

All periprocedural data are recorded in our electronic registries. These include the most commonly used behavioral scales.

The primary endpoint of the study was the evaluation of patients’ reactive behavior to facemask positioning for inhalational anesthesia induction. Secondary endpoints were as follows: (1) acceptance of midazolam/γ-cyclodextrin; (2) evaluation of premedication anxiolytic effect; (3) child’s behavior on separation from the parent; (4) the percentage of sevoflurane at which the child’s eyes closed upon induction of anesthesia; (5) time to eye closure at induction of anesthesia; (6) occurrence of delirium at the emergence from anesthesia; (7) evaluation of behavioral changes seven days after the procedure between the two groups.

Facemask acceptance was evaluated on a four-point scale (Mask Acceptance Scale, MAS) [23], with values indicating that the child is cooperative and accept the mask readily (MAS = 1), that they are lightly fearful and accept the mask with mild resistance (MAS = 2), that they accept it with a moderate struggle (MAS = 3), or that they resist strongly and must be restrained (MAS = 4). 

The degree of acceptance of the drug was rated on a four-point scale (Drug Acceptance Scale, DAS), with values indicating that the administered dose was accepted readily (DAS = 4), with disapproving grimaces (DAS = 3), with vocal disapproval (DAS = 2), or that it was completely rejected/spat out (DAS = 1). Midazolam/γ-cyclodextrin anxiolytic efficacy was evaluated starting 20 min after premedication administration with the Ramsay sedation scale (RSS) [24] which classifies level of awareness into six categories (awake: agitated, restless, or both (RSS = 1); awake: cooperative, oriented, and tranquil (RSS = 2); awake but responds to commands only (RSS = 3); asleep: brisk response to light glabellar tap or loud auditory stimulus (RSS = 4); asleep: sluggish response to light glabellar tap or loud auditory stimulus (RSS = 5); asleep: no response to glabellar tap or loud auditory stimulus (RSS = 6). Child’s behavior at the moment of separation from the parent just before induction of anesthesia was evaluated with a four-point Parental Separation Anxiety scale (PSAS), with values indicating a poor behavior with patient crying, need for restrain (PSAS = 1); fair: moderately afraid and crying, not quiet with reassurance (PSAS = 2); good: slightly afraid/crying, quiet with reassurance (PSAS = 3); excellent: unafraid and cooperative or asleep (PSAS = 4). 

The occurrence of ED at the end of the procedure was measured using the five items of the Pediatric Anesthesia Emergence Delirium scale (PAED) [25]. All items were scored on a 0–4 point scale as occurring not at all, just a little, quite a bit, very much, or extremely. Item scores were added together for a total score ranging from 0 to 20, with higher scores indicating more severe delirium. According to Sikich and Lerman, a PAED total score greater than 10 is regarded as the cut-off criteria for emergence agitation (EA) from anesthesia [26].

Maladaptive behavioral responses and developmental regression are also part of the dedicated section of the task force, which are evaluated by the children’s parents seven days after the procedure with the Post Hospitalization Behavior Questionnaire for Ambulatory Surgery (PHBQ-AS) [27].

### Statistical Analysis

Data are presented as mean (standard deviation) or median (interquartile range) for numerical data or N (%) for categorical or ordinal data. The normality distribution was assessed with Shapiro–Wilk test and visually by histograms. Continuous variables were compared with Student t or Mann–Whitney test; categorical variables with the Chi-square test or Fisher’s exact test in case of expected frequencies <5. Data analysis was performed using R (R Foundation for Statistical Computing, Vienna, Austria; version 4.1.2).

In a recent RCT by Zadrazil, midazolam/γ-cyclodextrin was compared to off-label conventional midazolam for premedication before elective surgery in children aged 2 to 8 years [23].

Midazolam/γ-cyclodextrin resulted in a mean of approximately 1.48 (SD 0.91) facemask acceptance score for inhalational anesthesia induction. Facemask acceptance score was evaluated on a four-point scale, with values indicating that the mask was accepted well and immediately (MAS = 1), after mild resistance (MAS = 2), after moderate struggle (MAS = 3), or with moderate force to overcome resistance (MAS = 4). Based on these results, we estimated a facemask acceptance score of 1.50 (SD 1) in the group premedicated with midazolam/γ-cyclodextrin compared to a facemask acceptance score of 2.20 (SD 1) in the Control group which did not receive any premedication. For independent samples *t*-test, with 100 patients, 50 per group would result in an effect size of 0.70 Cohens d.

## 3. Results

A total of 100 children (50 per group), aged 1 to 10 years, were included in this retrospective analysis. Most children were in the range age 1–4 years in both groups. 

Demographic, baseline characteristics, and duration of procedures were comparable between the two groups (Table 1).

Two (4%) children in the Control group were restless upon arrival at the MRI area.

As for the primary outcome, facemask positioning for induction of anesthesia was readily accepted by 29 (58%) children in the midazolam/γ-cyclodextrin group compared to 11 (22%) children in the Control group (*p* < 0.001). Sixteen (32%) children in the Control group needed to be restrained to mask positioning compared to five (10%) in the midazolam/γ-cyclodextrin group (*p* = 0.006) (Table 2).

Table 3 shows a significant oral acceptance of premedication: 31 (62%) children accepted it readily and 15 (30%) children accepted it with facial grimaces. The remaining 8% accepted the drug with vocal disapproval or completely rejected and spat it out. 

Regarding the anxiolytic effect of midazolam/γ-cyclodextrin, 30 min after drug administration, more than 90% of children showed RSS scores between 2 and 4: 37 (74%) children displayed a RSS score of 2, and appeared awake, cooperative, oriented, and tranquil. Three (6%) of them appeared agitated or restless (Table 4).

At the moment of separation from the parent, 38 (76%) premedicated children were unafraid and cooperative or asleep compared to 12 (24%) children in the Control group (*p* < 0.001). None of the premedicated children cried nor needed to be restrained compared to nine (18%) children in the Control group (*p* < 0.001) (Table 5).

A shorter time as well as a lower percentage of sevoflurane were needed for eye-closure at induction of anesthesia (*p* < 0.001) and for anesthesia maintenance (*p* < 0.037) (Table 6).

At emergence from anesthesia, 23 (46%) children in the premedicated group compared to 33 (66%) children (*p* = 0.043) in the Control group showed transient agitation and delirium as defined by a PAED score ≥ 10. More than 70% of children in both groups were aged 1–4. It was completely resolved within 20 min at discharge from PACU in both groups.

None of the patients experienced respiratory or hemodynamic complication during the procedure, and no adverse events related to the study medication were observed.

As regards behavioral changes, none of the parents reported new onset of postoperative maladaptive behavior at 7 days from the procedure, as evaluated by the PHBQ-AS (Table 6).

## 4. Discussion

MRI is a primary investigative tool for a wide range of pediatric medical conditions as it provides a detailed anatomical description not involving the use of ionizing radiations. Despite technological improvement, image acquisition requires an extended period of time and children are required to remain immobile in an unfamiliar and noisy environment.

To children who are too old to fall asleep using feeding and wrapping or too young to cooperate, or those facing behavioral or learning difficulties, non-pharmacological and pharmacological support may often be necessary.

To encourage a gentle approach to anesthesia induction, a range of non-pharmacological interventions such as parental presence or distraction techniques (e.g., clown or music therapy, videos) have emerged as adequately effective in reducing preoperative anxiety [10].

However, addressing the different etiological factors of anxiety, like age, autistic temperament, previous hospitalizations, family context, and socioeconomic status, requires specific psychological skills and professional profiles and can be time-consuming in daily clinical practice [28].

So, anxiolytics are often favored, especially outside the conventional operating room setting.

Midazolam, given its short half-life, has traditionally been a common choice in the pediatric population. However, when used as a single agent, it may not effectively control excessive movements, even during non-painful procedures such as MRI scans [14].

In the EU, the lack of specific labelling for orally administered anxiolytic-sedative drugs has led to the extended use of the intravenous formulation of midazolam for oral, nasal, or rectal administration as well. However, this practice involves an off-label route of administration without validated testing of bioavailability. Additionally, this method can contribute to poor compliance or reluctance in children due to potential irritation (nasal administration) or the bitter taste (oral administration). 

The recent licensing of midazolam/γ-cyclodextrin in Europe has provided an alternative for oral administration of midazolam.

In our study, midazolam combined with a γ-cyclodextrin for premedication in children undergoing elective MRI showed high levels of acceptance and allowed easier and less traumatic management of induction of anesthesia and awakening. It was readily accepted by 62% children, but 4% completely rejected and spat it out. Two of the four children with lower DAS scores, related to a poor acceptance of the drug, had experienced previous hospitalizations. Midazolam γ-cyclodextrin demonstrated a safe pharmacodynamic profile, and no adverse reactions were detected.

Population pharmacokinetic profile was evaluated by Guitted et al. from single-dose studies in a total of 49 participants, including 12 healthy adults [29] and 37 pediatric patients [30]. Overall, pharmacokinetic parameters of both midazolam and its primary active metabolite (γ-hydroxymidazolam) were comparable to that of orally administered intravenous midazolam solutions described in existing literature. The study reported a bioavailability value of 39.4% with a broad standard deviation, comparable to previous findings for orally administered drugs and for oral administration of conventional midazolam, both in adult and pediatric patients [31,32]. This confirms the well-known inter-individual variability typical of orally administered drugs and suggests that more than cyclodextrins, it is the pre-systemic metabolism by cytochrome P_450_ enzyme (CYP3A) that affects the bioavailability of midazolam, regardless the type of formulation [33,34]. In a phase 2 study involving 37 pediatric patients aged from 6 months to 17 years, a single 0.30 mg/Kg dose of midazolam/γ-cyclodextrin administered before anesthesia proved effective in achieving satisfactory sedation in 78% of the subjects within about 30 min, with comparable results across all age groups [35]. Sedation response rates obtained with 0.30 mg/Kg midazolam/γ-cyclodextrin were not significantly different to those reported in historical studies with a similar dose of 0.25 mg/Kg nor higher doses of 0.5, 0.75, 1.0, and 1.5 mg/Kg oral midazolam (78% vs. ~80–90%) (*p* = 0.94) [35]. Moreover, at a dose of 0.25 mg/Kg, sedation response rates were comparable whatever the types of formulation: midazolam/γ-cyclodextrin, commercial midazolam syrup, and hospital preparations of midazolam (*p* = 0.32) [35]. Our findings align with a recent randomized controlled trial conducted by Zadrazil et al., where they compared midazolam/γ-cyclodextrin with conventional midazolam as premedication in 80 children, aged 2 to 8 years, scheduled for elective surgery [23]. The level of anxiety was assessed 30 min later, at the time of anesthesia induction, by a modified Yale Preoperative Anxiety Scale-short form (mYPAS), and the authors found similar efficacy in reducing preoperative anxiety without significant differences between the two groups. However, while the midazolam/γ-cyclodextrin dose was accepted by all children, conventional midazolam was rejected by 15% of the children [23].

This suggests that the novel formulation may have advantages in terms of acceptance or tolerance compared to conventional midazolam, potentially contributing to better compliance among pediatric patients.

In agreement with the literature, we found lower levels of anxiety in the midazolam/γ-cyclodextrin group compared to children who were not premedicated, as evidenced by lower reported RSS scale scores and better acceptance of mask positioning during anesthesia induction.

Additionally, our study highlighted the positive impact of midazolam/γ-cyclodextrin on children’s behavior at the moment of separation from their parents, with most children being unafraid, cooperative, or asleep.

In a cohort study examining four parental presence clinical scenarios, in which parents and children were grouped based on their preoperative levels of anxiety, Kain et al. showed that the presence of the parent during induction of anesthesia does not always have an anxiolytic effect on the child: an anxious child benefits from the presence of a parent only if the parent is calm, and on the contrary, the presence of an overly anxious parent increases anxiety in a calm child [25].

In our study, all children were separated from the parent just before entering the MRI room, but because parental level of anxiety was not assessed, we are unable to judge whether the high rate of uncooperative or crying children in the Control group could have benefited from the presence of a calm parent during induction of anesthesia.

Studies evaluating the effects of premedication on anesthetic agents’ requirements in pediatric patients have found a 38% reduction in sevoflurane median effective concentration (EC_50_) with clonidine premedication [36], and a 17% and 21% sevoflurane EC_50_ reductions after oral midazolam and intranasal dexmedetomidine, respectively [37].

Sevoflurane is a preferred inhalational anesthetic for inducing anesthesia in children due to its non-pungent nature and low blood solubility, enabling rapid attainment of the necessary level of unconsciousness. It is extensively utilized in pediatric patients undergoing MRI procedures, exhibiting a high success rate of around 92% whether the patient is on mechanical ventilation or breathing spontaneously [38].

In this study, we observed that a lower Fi_Sevo_ was needed to obtain eye-closure in the midazolam/γ-cyclodextrin group compared to non-premedicated children. Gradual increments in the concentration of sevoflurane likely allowed for precise adjustments, leading to a smoother transition into the desired anesthesia state.

Conversely, non-premedicated children could have been less likely to receive the intended gradual administration of sevoflurane: their lack of cooperation and irascibility possibly led to the rapid attainment of higher concentrations to achieve anesthesia.

Despite its efficacy, the quality of emergence from sevoflurane anesthesia raises concerns as it may sometimes be complicated by transient agitation/delirium [39].

In our study, in the immediate post-anesthesia phase, we found that the number of children with agitation (PAED score ≥ 10) was significantly higher in the Control group. 

The reported prevalence of this disorder in the literature varies widely, ranging between 20% and 80%, and this discrepancy in prevalence rates can be attributed to different descriptive terms used, such as EA and ED, as well to variations in the monitored time intervals after anesthesia [7].

The precise cause of this phenomenon is not entirely understood, but several factors related to anesthesia, the patient, and the surgical procedure are recognized as predisposing factors. Interestingly, the patient population selected in our study is advantageous as it allows for the exclusion of pain as a major contributing factor to the incidence of EA [8].

In our study, we observed that children displayed confusion through moaning, restlessness, involuntary physical activity and thrashing about in the bed, without the need for pharmacological or physical restraint.

Sikich and Lerman suggested a cutoff criterion of a PAED total score greater than 10 to define EA from anesthesia [26], yet various investigators may employ different cutoffs for determining the presence of EA. Children with ED in our study presented a median PAED score of 12, without significant differences between the two groups. 

Despite the fact that no scale can distinguish EA from ED, the proximity of our PAED scores to the threshold value of 10 could be more suggestive of a transient form of post-anesthetic agitation [40], especially considering the subsequent disappearance of these symptoms within a few minutes upon admission to the PACU. Furthermore, the absence of behavioral changes at the one-week point aligns with the findings of Kain et al., indicating that the observed agitation after anesthesia might indeed be a transient phenomenon rather than a persistent behavioral change [9]. The lack of differences between the two groups in EA/ED at discharge to the ward and of behavioral changes one week later would be interesting to relate to eventual successive sedations/anesthesia procedures. Often, MRI controls are required in pediatric patients, and previous experiences can influence stress disorders.

This study presents some limitations. The first is due to the retrospective nature of the study. One significant limitation is the lack of evaluation of parental anxiety, which could have affected the anxiety experienced by children. While examining this factor would be beneficial, it presents challenges due to the complex dynamics that may be present in certain familial contexts. The third is given by the choice of the Ramsay scale which, although widely used, is more likely to better define deeper levels of sedation. A favorable aspect of the study is the implementation of several scales of monitoring about behavioral changes, which allowed us to more correctly analyze subjective patterns. According to us, it would be recommendable to be used with this approach in order to guarantee better performances and control of the quality of sedation/anesthesia on pediatric patients.

## 5. Conclusions

Administering anesthesia to children can be very challenging, as it can be affected by various factors like age, personality, history of prior medical procedures, family dynamics, and the expertise of medical practitioners [41]. It is crucial for anesthesiologists to focus on alleviating children’s anxiety by promoting a nurturing setting, calming their apprehensions, and developing a relationship with the medical staff.

Combination of midazolam with γ -cyclodextrin offers favorable acceptance and satisfactory anxiolysis. This is reflected in the decreased requirement for anesthetics and potentially contributes to reducing the onset of EA. Future prospective randomized studies also investigating age-related pharmacokinetic differences to optimize medication dosing strategies could further elucidate the safety profile and confirm the clinical advantages of this new formulation of midazolam. In addition, including the examination of how various geographical and cultural family backgrounds influence anxiety levels and behavioral reactions in children in research studies could enhance our understanding of their impact and the effectiveness to treatment. This could ultimately lead to more personalized and effective interventions.

## Figures and Tables

**Table 1:** Baseline characteristics.

Characteristic	Midazolam/γ-Cyclodextrin Group (N = 50)	Control Group (N = 50)	*p* Value
Age	4 (2, 5)	4 (2, 6)	0.628
Age 1–4 years	34 (68)	29 (58)	0.300
Age 5–8 years	12 (24)	16 (32)	0.372
Age 9–10 years	4 (8)	5 (10)	0.999
Gender			0.105
Female	25 (50)	17 (34)	
Male	25 (50)	33 (66)	
Weight, kg	15 (12, 22)	15 (11, 23)	† 0.909
ASA status 1	18 (36)	13 (26)	0.279
ASA status 2	31 (62)	32 (64)	0.835
ASA status 3	1 (2)	5 (10)	0.204
Mild intellectual disability	3 (6)	3 (6)	>0.999
Moderate intellectual disability	2 (4)	3 (6)	>0.999
Previous hospitalizations	14 (28)	23 (46)	0.623
**Indication to MRI**			
Brain or spinal cord benign or malignant tumors	9 (18)	8 (16)	0.790
Extracranial malignant tumors	1 (2)	3 (6)	0.617
Cerebral venous thrombosis	0 (0)	2 (4)	0.494
Craniosynostosis	3 (6)	2 (4)	>0.999
Epilepsy	3 (6)	6 (12)	0.487
Spina bifida	2 (4)	3 (6)	>0.999
Autism spectrum disorder	3 (6)	5 (10)	0.715
Polymalformative syndrome	10 (20)	6 (12)	0.275
Growth restriction	16 (32)	9 (18)	0.105
Preterm birth	3 (6)	6 (12)	0.487

Demographic, baseline, and clinical characteristics of the study population. Data are presented as N (%) or median (25th to 75th IQR). *p* value corresponded to Chi Square test or Fisher’s exact test in case of expected frequencies <5. † *p* value corresponded to Wilcoxon rank sum test. ASA: American Society of Anesthesiologists. MRI: magnetic resonance imaging.

**Table 2:** Acceptance of facemask positioned for inhalational anesthesia induction.

	Midazolam/γ-Cyclodextrin Group (N = 50)	Control Group (N = 50)	*p* Value
Mask accepted readily	29 (58)	11 (22)	<0.001
Mask accepted with mild resistance	11 (22)	14 (28)	0.4888
Mask accepted with moderate struggle	5 (10)	9 (18)	0.249
Need to be restrained to mask positioning	5 (10)	16 (32)	0.006

Data are presented as N (%). *p* value corresponded to Chi Square test or Fisher’s exact test in case of expected frequencies <5.

**Table 3:** Acceptance of midazolam/γ-cyclodextrin premedication drug.

	Midazolam/γ-Cyclodextrin Group (N = 50)
Dose accepted readily	31 (62)
Dose accepted with facial grimaces	15 (30)
Dose accepted with vocal disapproval	2 (4)
Dose completely rejected or spat out	2 (4)

Data are presented as N (%).

**Table 4:** Evaluation of midazolam/γ-cyclodextrin anxiolytic efficacy 30 min after administration with the Ramsay sedation scale.

	Midazolam/γ-Cyclodextrin Group (N = 50)
Awake; agitated or restless or both	3 (6)
Awake; cooperative, oriented, and tranquil	37 (74)
Awake but responds to commands only	6 (12)
Asleep; brisk response to light glabellar tap or loud auditory stimulus	4 (8)
Asleep; sluggish response to light glabellar tap or loud auditory stimulus	0 (0)
Asleep; no response to glabellar tap or loud auditory stimulus	0 (0)

Data are presented as N (%).

**Table 5:** Child’s behavior at the moment of separation from the parent just before induction of anesthesia.

	Midazolam/γ-Cyclodextrin Group (N = 50)	Control Group (N = 50)	*p* Value
Unafraid and cooperative or asleep	38 (76)	12 (24)	<0.001
Slightly afraid/crying, quiet with reassurance	7 (14)	15 (30)	0.534
Moderately afraid and crying, not quiet with reassurance	5 (10)	15 (30)	0.012
Crying, need for restraint	0 (0)	9 (18)	<0.001

Data are presented as N (%). *p* value corresponded to Chi Square test or Fisher’s exact test in case of expected frequencies <5.

**Table 6:** Procedural and post-procedural clinical data.

Characteristic	Midazolam/γ-Cyclodextrin Group (N = 50)	Control Group (N = 50)	*p* Value
Duration of MRI, min	19 (17, 23)	23 (17, 31)	0.053
Heart rate at induction of inhalational anesthesia, bpm	102 (94, 112)	100 (89, 110)	0.120
SpO_2_ at induction of inhalational anesthesia	98 (98, 99)	98 (98, 99)	0.470
SpO_2_ at arousal from inhalational anesthesia	98 (97, 99)	98 (98, 99)	0.315
Highest EtCO_2_ during inhalational anesthesia	41 (38, 42)	39 (35, 41)	0.048
Percentage of sevoflurane for eye closure at induction of inhalational anesthesia	2 (2, 3)	8 (6, 8)	<0.001
Percentage of sevoflurane for maintenance of inhalational anesthesia	2 (2, 2)	2.5 (2, 2.5)	<0.001
Time to eye closure at induction of inhalational anesthesia	70 (90, 110)	90 (80, 170)	0.037
Children with emergence delirium at emergence from inhalational anesthesia	23 (46)	33 (66)	† 0.043
PAED score in children with ED	12 (11, 13)	12 (11, 14)	0.634
Children with ED 15 min after emergence from inhalational anesthesia	1 (2)	6 (12)	† 0.111
Children with ED at discharge from PACU	0 (0)	0 (0)	† >0.999
Parents ‘reported new onset of maladaptive behaviours at 7 days from the procedure evaluated with the PHBQ-AS	0 (0)	0 (0)	† >0.999

Data are presented as N (%) or median (25th to 75th IQR. *p* value corresponded to Wilcoxon rank sum test. † *p* value corresponded to Chi Square test or Fisher’s exact test in case of expected frequencies <5. Emergence delirium was defined as a PAED score ≥ 10. MRI: magnetic resonance imaging. SpO_2_: saturation of peripheral oxygen. EtCO_2_: end-tidal carbon dioxide. PAED: Pediatric Anesthesia Emergence Delirium. PACU: Post-Anesthesia Care Unit. ED: Emergence Delirium; PHBQ-AS: Post-Hospitalization Behavior Questionnaire for Ambulatory Surgery.

## Data Availability

Data is unavailable due to Institutional restrictions.
